# Serum albumin combined with high-density lipoprotein cholesterol as a novel marker to predict coronary heart disease: are their associations multiplicative or rateable?

**DOI:** 10.3389/fcvm.2025.1508972

**Published:** 2025-07-30

**Authors:** Li He, Sisi Chen, Xuan Zhu, Fang He

**Affiliations:** Department of Emergency, Wuhan Fourth Hospital, Wuhan, Hubei, China

**Keywords:** albumin, high-density lipoprotein cholesterol, coronary heart disease, albumin multiplied by high-density lipoprotein cholesterol, albumin–high-density lipoprotein cholesterol ratio

## Abstract

**Background:**

Coronary heart disease (CHD) is a leading cause of death and disability worldwide. Albumin (ALB) and high-density lipoprotein cholesterol (HDL-c) possess potential clinical application values. However, the relationship between ALB*HDL-c (AHM) and CHD in the general population has not been studied yet. Therefore, this study aims to investigate the association between the AHM and CHD.

**Methods:**

We conducted a retrospective study using data from 2,568 patients with a diagnosis of CHD from Wuhan Fourth Hospital. Patients with one or more major coronary artery or branch vessel stenosis ≥ 50% were included in the CHD group, while patients without CHD were enrolled in the control group. Logistic regression analysis was performed to determine the influence of AHM on CHD. The receiver operating characteristic (ROC) curve was constructed to analyze the predictive value of AHM for CHD.

**Results:**

A total of 1,824 enrolled patients (71.0%) were diagnosed with CHD. The mean age was 64.56 ± 10.08 years. Notably, the CHD group had a substantially lower median AHM than that of the control group (36.94 vs. 52.63), with a statistically significant difference (*P* < 0.05). Specifically, logistic regression demonstrated that AHM was an independent risk factor for CHD (OR = 0.903, 95% CI: 0.888–0.918) in identifying CHD. In ROC analysis, the area under the ROC curve (AUC) for AHM [0.808 (95% CI: 0.791–0.825, *P* < 0.001)] was larger than that for ALB, HDL-c, and ALB–HDL-c ratio (AHR), and the differences were statistically significant (*P* < 0.05). Additionally, the Gensini (GS) score was negatively correlated with AHM (*R* = −0.150, *P* < 0.001). AHM was significantly associated with multivessel CHD (OR = 0.903, 95% CI: 0.888–0.918), and ROC analysis showed an AUC of 0.639 for AHM in predicting multivessel CHD.

**Conclusion:**

AHM was significantly linked to an elevated risk of CHD. The lower the AHM level, the greater the CHD occurrence rate. AHM is associated not only with the occurrence of CHD but also with the severity of coronary artery stenosis. This underscores the crucial value of AHM in the discrimination and management of CHD.

## Introduction

With the transformation of social structure, population aging has been escalating year by year, accompanied by a rising trend in the prevalence and mortality of coronary heart disease (CHD). As of 2019, the global burden of CHD had reached approximately 197 million patients, making it one of the most prevalent cardiovascular diseases (CVD) and the second leading cause of death in China (1). From 1990 to 2017, the global age-standardized mortality rate for CHD increased by 20.6%, with the most substantial rises observed in Africa and other low- to middle-income nations (2). Projections indicate a persistent upward trend in CHD mortality. This disease imposes substantial physical, psychological, and socioeconomic burdens, adversely affecting individual well-being, family welfare, and healthcare expenditures. The early identification of risk factors and timely detection of high-risk individuals are pivotal for the implementation of effective preventive strategies and tailored interventions.

As our understanding of CHD pathogenesis deepens, there is an increasing focus on the multiple potential factors influencing CHD development. Well-recognized risk factors for CHD include hypertension, insulin resistance, dyslipidemia [elevated low-density lipoprotein cholesterol (LDL-c) or reduced high-density lipoprotein cholesterol (HDL-c)], smoking, excessive alcohol intake, overweight/obesity, psychological stress, and advanced age (3–5). With the exception of age (a non-modifiable risk factor), other determinants can be effectively managed via lifestyle adjustments, stress reduction, and pharmacologic interventions to slow CHD progression (6, 7). HDL-c remains the most significant protective factor against CHD among parameters measured in routine lipid panels (8). HDL-c plays a central role in reverse cholesterol transport, shuttling extrahepatic cholesterol to the liver for catabolism, exerting functions of inhibiting LDL oxidation and platelet activation, and serving as a key anti-atherosclerotic lipoprotein (9). HDL-c has long been considered inversely related to CHD risk. In other words, low levels of HDL-c are a risk factor for CHD, which has been confirmed by several previous studies (10, 11).

Albumin (ALB), the most abundant water-soluble protein in plasma, plays a pivotal role in maintaining osmotic pressure, functioning as an antioxidant, acting as a biological buffer, and mediating transport of endogenous and exogenous substances. It exhibits both antioxidant and anti-inflammatory activities (12). ALB serves as a key biomarker for nutritional status and chronic inflammatory states, with reduced levels commonly linked to chronic pathologies and malnutrition (13). Numerous observational studies and meta-analyses have demonstrated a negative association between serum albumin levels and cardiovascular outcomes (14). Numerous observational investigations and meta-analytic syntheses have consistently evidenced an inverse association between serum ALB concentrations and cardiovascular event rates (15, 16). Accumulating evidence indicates that hypoalbuminemia is associated with heightened risks of major adverse cardiovascular events (MACE) and in-hospital mortality (16). Oduncu et al. (17) demonstrated that admission serum ALB levels strongly predicted clinical outcomes in ST-segment elevation myocardial infarction (STEMI) patients treated with percutaneous coronary intervention (PCI). Hypoalbuminemia (lower serum ALB concentration) is closely associated with elevated cardiovascular risk. Pooled data from meta-analyses indicate that each 2.5 g/L decrease in serum ALB is associated with a 24%–56% higher mortality odds ratio, encompassing both all-cause and cardiovascular-specific mortality outcomes (18). Serum ALB concentration strongly and independently predicts in-hospital all-cause mortality, demonstrating a negative association with mortality (19). Numerous prior studies have established that low serum ALB is a robust predictor of CHD. A representative analysis has demonstrated a significant correlation between serum ALB levels and CHD risk, whereby lower ALB concentrations are associated with an elevated risk of CHD (20).

Both ALB and HDL-c correlate with the onset and progression of CHD, yet their combined relationship with CHD remains underexplored. Two new combined indicators of nutritional status and lipid metabolism, namely, ALB multiplied by HDL-c (AHM) and ALB–HDL-c ratio (AHR), may hold importance in CHD prediction. These findings, by identifying novel predictive biomarkers, may enable more timely clinical interventions for CHD patients.

## Method

### Study design and subjects

A total of 2,885 patients who underwent coronary angiography (CAG) at Wuhan Fourth Hospital between August 2022 and August 2023 were enrolled, all of whom complied with the following inclusion and exclusion criteria. The inclusion criteria were as follows: (1) CHD patients who met the diagnostic criteria of the updated guidelines for the treatment of CHD and (2) those aged >18 years old. The exclusion criteria were as follows: (1) patients with diseases that may affect HDL-c or ALB metabolism, including severe kidney damage; (2) patients with severe liver diseases such as liver cirrhosis and biliary obstructive diseases; (3) patients with severe cardiac membrane disease, severe heart failure, and congenital CHD; (4) patients with malignant tumors and autoimmune diseases; and (5) patients with other lesions such as cardiogenic shock and severe infection. According to CHD diagnostic criteria, participants were allocated to either the CHD group or the control group. This study adhered to the principles of the Declaration of Helsinki and obtained ethical approval from the Ethics Committee of Wuhan Fourth Hospital (Ethics Approval No. KY2023-117-01). Given its retrospective nature, the Ethics Committee waived the requirement for informed consent, and all personal identifiers were removed from the dataset.

### Date collection and definitions

Sociodemographic data such as gender, age, history of hypertension, type 2 diabetes mellitus (T2DM), and smoking were collected through an independent digital patient record system. Hypertension was defined as systolic blood pressure (SBP) ≥ 140 mmHg and/or diastolic blood pressure (DBP) ≥ 90 mmHg or current use of hypotensive drugs (21). The diagnosis of diabetes mellitus was based on one or more of the following: (1) self-reported history of diabetes mellitus; (2) hypoglycemic drugs; (3) fasting blood glucose (FBG) ≥7.0 mmol/L; and (4) blood ≥11.1 mmol/L 2 h after oral glucose tolerance test (OGTT) (22).

All the laboratory exam indexes related to the patients were collected, including serum alanine aminotransferase (ALT), aspartate aminotransferase (AST), ALB, γ-glutamyltransferase (GGT), TC, TG, HDL-c, LDL-c, creatinine (Cr), uric acid (UA), blood glucose (GLu), glycated hemoglobin (HbA1c), and other indicators. Fasting venous blood samples were collected the morning after admission, from which all indicators were assayed, and samples were sent to the hospital laboratory for analysis.

### Diagnosis and grouping of the severity of coronary artery disease

CAG is the gold standard for the diagnosis of CHD. The patients were classified into the CHD group if CAG revealed ≥50% stenosis in one or more major coronary arteries (including their branches), whereas those without CHD were assigned to the non-CHD control group. The patients were classified as having single-vessel CHD if a single major coronary artery (with ≥50% stenosis) was involved and multivessel CHD if two or more major coronary arteries showed significant stenosis. The Gensini (GS) score, a validated quantitative measure, was used to evaluate coronary lesion severity, with scores calculated based on the anatomical location and percentage of luminal stenosis. Coronary artery stenosis severity was assessed using the GS score, whereby the score for each coronary lesion was determined by multiplying the anatomical site score (across seven predefined coronary segments) by the stenosis grade score (six grades of luminal narrowing). The total GS was computed as the aggregate of all individual lesion scores (23).

### Statistical analysis

Statistical analyses were performed using SPSS 27.0. The Kolmogorov–Smirnov test (K–S test) was employed to assess the normality of data distribution. Normally distributed continuous variables were compared using the independent samples *t*-test and presented as mean ± standard deviation. Non-normally distributed variables were analyzed via the Mann–Whitney *U* test and reported as median [interquartile range (IQR)]. Categorical variables were reported as percentages, and intergroup differences between the CHD and control groups were evaluated using the Pearson chi-square test. For comparisons across three groups, one-way ANOVA was performed, followed by Bonferroni-corrected *post hoc* tests. Spearman's rank correlation coefficient was calculated to assess the relationship between serological biomarkers and GS score, reflecting coronary stenosis severity. Logistic regression modeling was employed to identify independent risk factors for CHD and predict the severity of coronary artery stenosis, with model performance evaluated via AUC-ROC analysis. Variables significant at *P* < 0.10 in univariate analyses, along with established risk factors, were entered into a multivariate logistic regression model. To address multicollinearity, ALB and HDL-c were omitted from the regression models. Receiver operating characteristic (ROC) curves were constructed, and area under the curve (AUC) values were computed to evaluate predictive accuracy for CHD. Model performance was visualized using GraphPad Prism 8.0. Statistical significance was set at *P* < 0.05 (two-tailed).

## Results

### General data analysis

**Figure 1:**
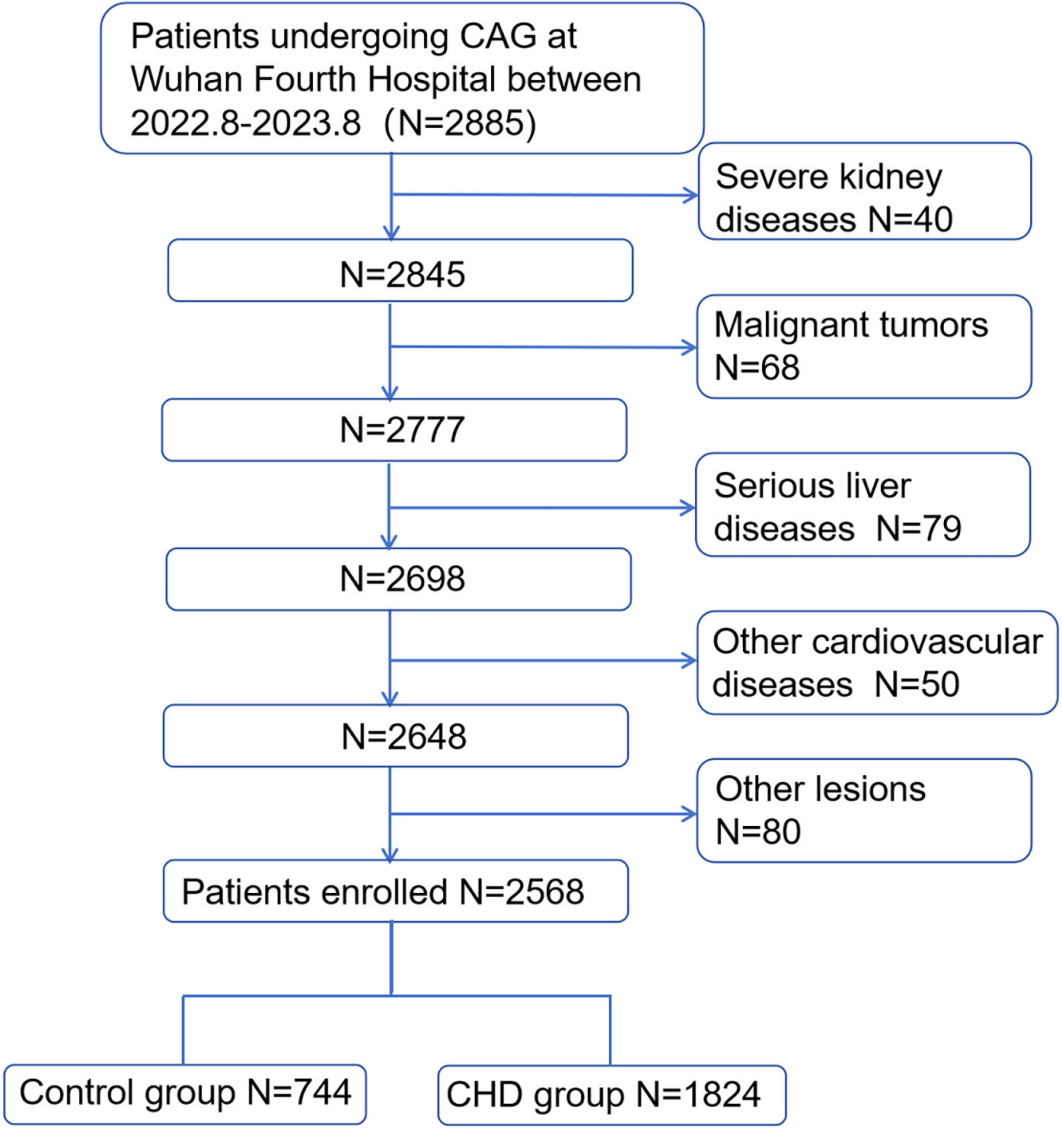
Population flowchart of enrolled patients. CHD, coronary heart disease; CAG, coronary angiography.

A total of 2,885 patients underwent CAG, of whom 2,568 met the inclusion criteria and were enrolled in the study. Based on CHD diagnostic criteria, these patients were categorized into a CHD group (*n* = 1,824) and a control group (*n* = 744) (Figure 1). The mean age was 64.56 ± 10.08 years. The CHD group exhibited significantly higher proportions of male sex, advanced age, smoking history, hypertension, T2DM, and antilipidemic drug use, along with elevated serum levels of ALT, GGT, HbA1c, Glu, Cr, AHR, LDL-c, AST, TG, and UA, compared with the control group (all *P* < 0.05). Conversely, serum levels of ALB, HDL-c, and TC were significantly lower in the CHD group than those in the control group (all *P* < 0.05). Notably, the CHD group had a substantially lower median AHM than that in the control group (36.94 vs. 52.63), with a statistically significant difference (*P* < 0.05) (Table 1).

**Table 1:** Clinical and laboratory characteristics of patients.

Parameters	Control group, *N* = 744	CHD group, *N* = 1,824	*P*
Sex, male (*n*%)	312 (41.9)	1,079 (59.1)	<0.001
Age (year)	60 (53, 67)	66 (59, 71)	<0.001
Hypertension [*n* (%)]	408 (54.8)	1,362 (74.6)	<0.001
T2DM [*n* (%)]	157 (21.1)	690 (37.8)	<0.001
Smoking [*n* (%)]	123 (16.5)	482 (26.4)	<0.001
Drinkers (*n*%)	57 (7.6)	194 (10.6)	0.021
Antilipidemic drug therapy (*n*%)	590 (79.3)	1,708 (93.6)	<0.001
HDL-c (mmol/L)	1.21 (1.03, 1.43)	0.92 (0.78, 1.12)	<0.001
LDL-c (mmol/L)	2.51 (1.92, 3.16)	2.37 (1.72, 3.05)	<0.001
TG (mmol/L)	1.39 (0.99, 2.03)	1.49 (1.04, 2.22)	0.001
TC (mmol/L)	4.38 (3.71, 5.14)	4.14 (3.35, 4.95)	<0.001
ALT(mmol/L)	19 (14, 29)	21 (15, 30)	0.037
AST (mmol/L)	22 (18, 26)	22 (18, 30)	0.005
ALB (g/L)	43.53 ± 3.54	40.03 ± 3.66	<0.001
GGT (U/L)	22 (16, 38)	25 (18, 40)	<0.001
HbA1c (mmol/L)	5.8 (5.4, 6.4)	6.30 (5.70, 7.40)	<0.001
Glu (mmol/L)	5.76 (5.05, 7.13)	6.29 (5.30, 8.41)	<0.001
Cr (μmol/L)	67.85 (57.28, 79.73)	74.20 (62.02, 88.40)	<0.001
UA (μmol/L)	338 (276, 407)	353 (288, 425)	0.001
AHM	52.63 (44.11, 62.61)	36.94 (30.22, 45.29)	<0.001
AHR	0.49(0.41, 0.62)	0.55(0.45, 0.74)	<0.001

AHR, albumin–high-density lipoprotein cholesterol ratio; AHM, albumin multiplied by high-density lipoprotein cholesterol; ALB, albumin; ALT, alanine aminotransferase; AST, aspartate aminotransferase; CHD, coronary heart disease; Cr, creatinine; GGT, glutamyl transpeptidase; GLU, glucose; HbA1c, glycosylated hemoglobin; HDL-c, high-density lipoprotein cholesterol; LDL-c, low-density lipoprotein cholesterol; TC, total cholesterol; TG, triglycerides; T2DM, type 2 diabetes mellitus; UA, uric acid.

### Logistic regression analysis

Multivariable logistic regression analysis was conducted to identify independent risk factors for CHD, following univariate screening of all candidate variables. Univariate logistic regression analysis showed that there was statistical significance in age, sex, hypertension, T2DM, smoking, LDL-c, TG, TC, ALT, AST, HbA1c, GLU, Cr, UA, AHR, and AHM (*P* < 0.05). After adjusting for confounding factors, multiple regression analysis showed that AHM as a continuous variable remained an independent risk factor for CHD (OR = 0.903, 95% CI: 0.888–0.918, *P* < 0.001). Therefore, AHM was an independent predictor of CHD (Table 2).

**Table 2:** Univariate and multivariate logistic regression analyses for CHD patients.

	Univariate		Multivariate	
	OR (95% CI)	*P*	OR (95% CI)	*P*
Sex	0.499 (0.419–0.593)	<0.001	0.552 (0.387–0.786)	0.001
Age (year)	1.040 (1.031–1.049)	<0.001	1.029 (1.013–1.045)	<0.001
Hypertension	1.813 (1.456–2.259)	<0.001	2.058 (1.513–2.799)	<0.001
T2DM	1.434 (1.054–1.953)	<0.001	1.517 (1.055–2.181)	0.025
Smoking	2.428 (2.030–2.903)	<0.001	1.294 (0.800–2.093)	0.293
Drinkers	2.275 (1.863–2.778)	0.022	0.631 (0.327–1.218)	0.170
LDL-C	0.859 (0.786–0.940)	0.001	0.281 (0.180–0.441)	<0.001
TG	1.072 (1.007–1.140)	0.029	0.655 (0.567–0.756)	<0.001
TC	0.853 (0.792–0.918)	<0.001	4.096 (2.651–6.328)	<0.001
ALT	1.005 (1.001–1.009)	0.017	0.997 (0.988–1.006)	0.503
AST	1.013 (1.008–1.018)	<0.001	0.910 (0.844–0.980)	0.013
GGT	1.001 (1.000–1.003)	0.137		
HbA1c	1.350 (1.232–1.479)	<0.001	1.048 (0.901–1.219)	0.543
GLU	1.116 (1.079–1.154)	<0.001	0.984 (0.931–1.041)	0.581
Cr	1.013 (1.009–1.018)	<0.001	1.001 (0.996–1.006)	0.640
UA	1.001 (1.000–1.002)	0.002	0.999 (0.997–1.000)	0.118
AHM	0.918 (0.911–0.926)	<0.001	0.903 (0.888–0.918)	<0.001
AHR	2.527 (1.889–3.379)	<0.001	82.685(3.677–1,859.302)	0.005

After adjusting for confounding factors: sex, age, hypertension, diabetes mellitus, smoking, LDL, TC, TG, ALT, AST, GGT, HbA1c, GLU, Cr, UA.

AHR, albumin–high-density lipoprotein cholesterol ratio; AHM, albumin multiplied by high-density lipoprotein cholesterol; ALB, albumin; ALT, alanine aminotransferase; AST, aspartate aminotransferase; CHD, coronary heart disease; Cr, creatinine; GGT, glutamyl transpeptidase; GLU, glucose; HbA1c, glycosylated hemoglobin; HDL-c, high-density lipoprotein cholesterol; LDL-c, low-density lipoprotein cholesterol; TC, total cholesterol; TG, triglycerides; T2DM, type 2 diabetes mellitus; UA, uric acid.

### ROC analysis

**Figure 2:**
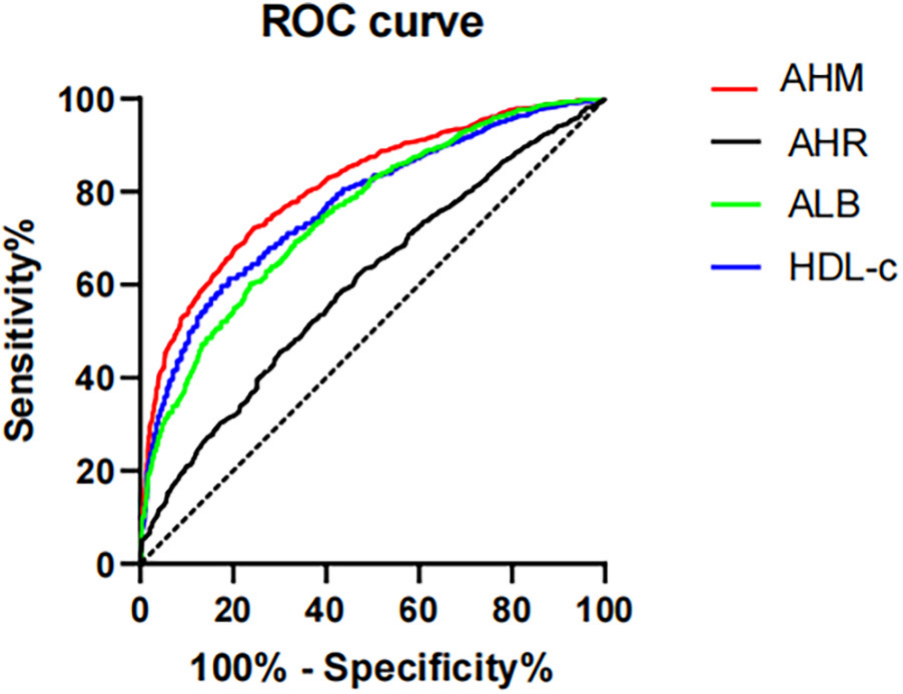
Receiver operating characteristics curves of AHM and other indexes for identifying CHD. AHM, albumin multiplied by high-density lipoprotein cholesterol; AHR, albumin–high-density lipoprotein cholesterol ratio; ALB, albumin; HDL-c, high-density lipoprotein cholesterol; CHD, coronary heart disease.

To assess the predictive accuracy of HDL-c, AHM, and ALB for CHD, ROC curve analysis was performed (Figure 2). HDL-c showed a cutoff value of 0.990, with 81.9% sensitivity, 59.8% specificity, and a Youden index of 0.417 (AUC = 0.770, 95% CI: 0.751–0.789, *P* < 0.001). ALB had an optimal cutoff of 40.950, yielding 76.1% sensitivity, 60.3% specificity, and an AUC of 0.752 (95% CI: 0.732–0.772, *P* < 0.001). AHM demonstrated the highest discriminative ability (AUC = 0.808, 95% CI: 0.791–0.825, *P* < 0.001) at a cutoff of 43.796 (75.7% sensitivity, 71.6% specificity), whereas AHR had the lowest AUC (0.694, 95% CI: 0.673–0.716, *P* < 0.001) at a cutoff of 0.263 (47.6% sensitivity, 81.6% specificity). These findings indicate that AHM outperforms HDL-c and AHR in predicting CHD (Table 3).

**Table 3:** Receiver operating characteristics curves of AHM and other indexes for identifying CHD.

	ALB	HDL-c	AHM	AHR
Critical value	40.950	0.990	43.796	43.959
Sensitivity	0.761	0.819	0.757	0.476
Specificity	0.603	0.598	0.716	0.819
AUC	0.752 (0.732–0.772)	0.770 (0.751–0.789)	0.808 (0.791–0.825)	0.694 (0.673–0.716)
*P*-value	<0.001	<0.001	<0.001	<0.001
Youden index	0.364	0.417	0.473	0.295

AHR, albumin–high-density lipoprotein cholesterol ratio; AHM, albumin multiplied by high-density lipoprotein cholesterol; ALB, albumin; HDL-c, high-density lipoprotein cholesterol.

### Association between AHM and CHD severity

**Figure 3:**
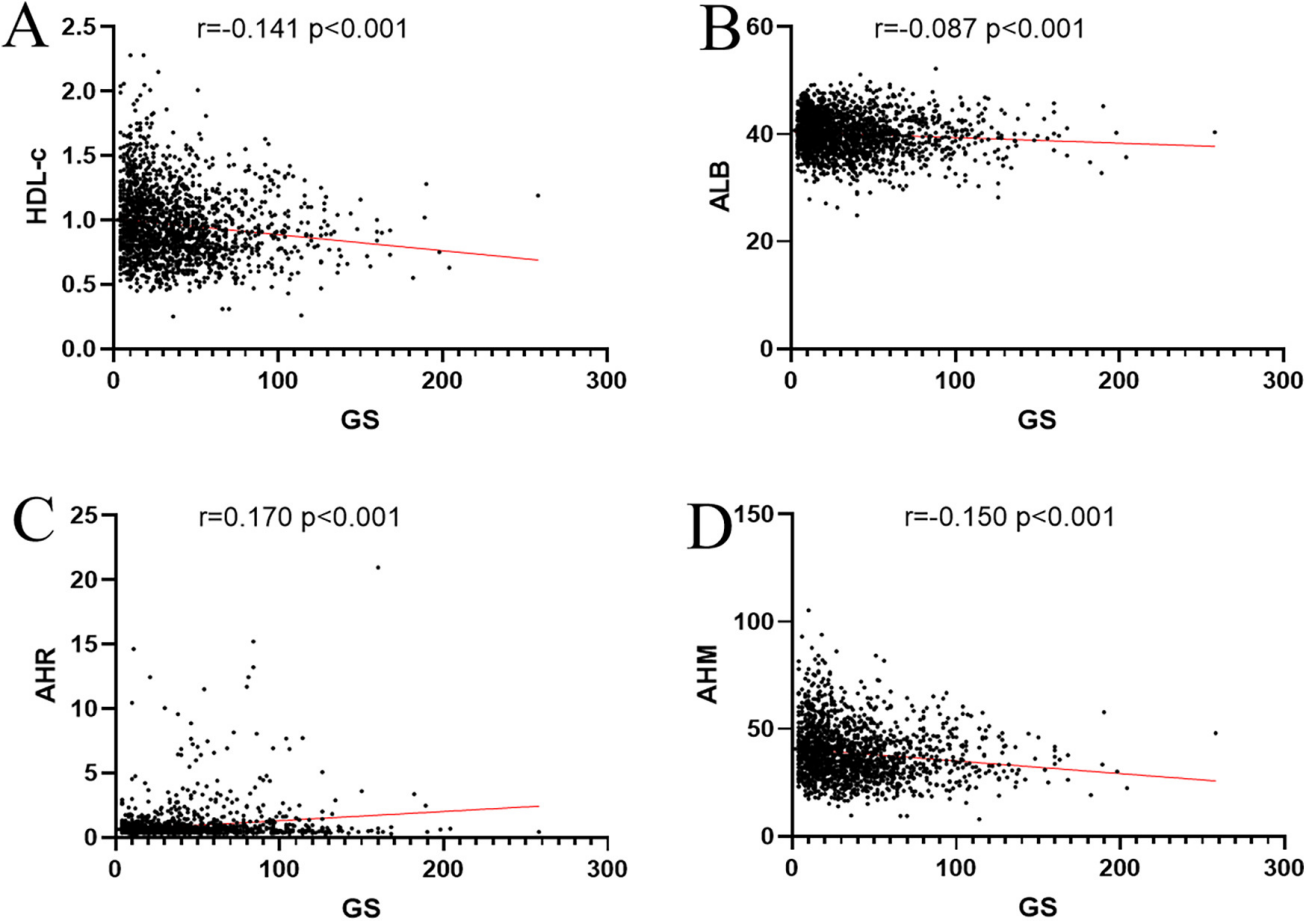
Correlation coefficient between AHM, ALB, HLD-c, AHR, and Gensini score. **(A)** HDL-c. **(B)** ALB. **(C)** AHR. **(D)** AHM. AHM, albumin multiplied by high-density lipoprotein cholesterol; AHR, albumin–high-density lipoprotein cholesterol ratio; ALB, albumin; HDL-c, high-density lipoprotein cholesterol; GS, Gensini score.

**Figure 4:**
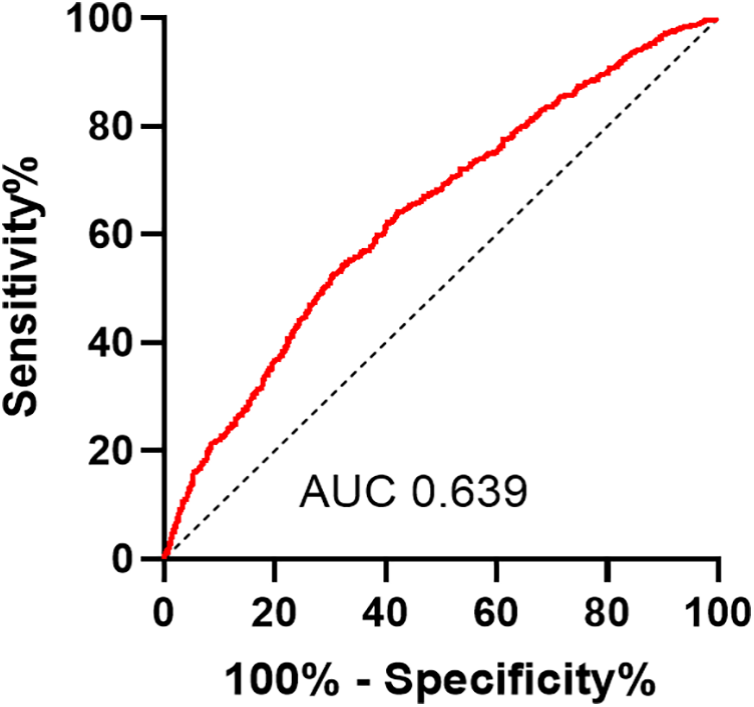
Receiver operating characteristic curves of AHM for identifying multivessel CHD patients. AHM, albumin multiplied by high-density lipoprotein cholesterol; CHD, coronary heart disease.

We used Spearman to analyze the correlation between influence factors and the GS score in CHD. As shown in Table 4, the GS score was negatively correlated with sex HDL-c, ALB, and AHM (Figure 3). It was positively correlated with LDL-c, TC, AST, HbA1c, GLU, Cr, smoking, and T2DM. It was not associated with TG, ALT, GGT, UA, age, drinkers, and hypertension. The CHD patients were categorized into two groups, single-vessel CHD (*n* = 645) and multivessel CHD (*n* = 1,179). As shown in Table 5, logistic regression models were constructed to show that the AHM was significantly related to CHD severity before and after multivariate adjustment (OR = 0.960, 95% CI: 0.952–0.968, *P* < 0.001). When the AHM was analyzed as a continuous variable, it was significantly associated with multivessel CHD (OR = 0.979, 95% CI: 0.968–0.991, *P* < 0.001). The ROC curve for multivessel CHD and AHM is shown in Figure 4. The cutoff value of AHM was 28.856, and the area under the ROC curve was 0.639 (95% CI: 0.612–0.665, *P* < 0.001) for predicting multivessel CHD. The results show that the higher the AHM is, the more severe the coronary lesions become.

**Table 4:** Correlation coefficient between different factors and Gensini score.

	Gensini score	
	*R*	*P*
HDL-c	−0.141**	<0.001
LDL-c	0.179**	<0.001
TG	0.042	0.070
TC	0.123**	<0.001
ALT	0.025	0.290
AST	0.162**	<0.001
ALB	−0.087**	<0.001
GGT	−0.013	0.567
HbA1c	0.166**	<0.001
GLU	0.127**	<0.001
Cr	0.081**	0.001
UA	0.037	0.121
Sex	−0.162**	<0.001
Age (year)	−0.037	0.117
Smoking	0.123**	<0.001
Drinkers	0.019	0.414
Hypertension	0.032	0.175
T2DM	0.121**	<0.001
AHM	−0.150**	<0.001
AHR	0.170**	<0.001

*P < 0.05.

**P < 0.01.

AHR, albumin–high-density lipoprotein cholesterol ratio; AHM, albumin multiplied by high-density lipoprotein cholesterol; ALB, albumin; ALT, alanine aminotransferase; AST, aspartate aminotransferase; CHD, coronary heart disease; Cr, creatinine; GGT, glutamyl transpeptidase; GLU, glucose; GS, Gensini score; HbA1c, glycosylated hemoglobin; HDL-c, high-density lipoprotein cholesterol; LDL-c, low-density lipoprotein cholesterol; TC, total cholesterol; TG, triglycerides; T2DM, type 2 diabetes mellitus; UA, uric acid.

**Table 5:** Association between AHM and CHD severity.

Variables	Multivessel CHD	
	OR (95% CI)	*P*-value
Crude model	0.960 (0.952–0.968)	<0.001
Model 1	0.968 (0.959–0.976)	<0.001
Model 2	0.979 (0.968–0.991)	<0.001

The crude model was not adjusted.

Model 1 was adjusted for sex, smoking, alcohol consumption, hypertension, T2DM, and antilipidemic drug therapy.

Model 2 was further adjusted for sex, smoking, alcohol consumption, hypertension, T2DM, antilipidemic drug therapy, AST, Glu, HbA1c, Cr, and AHR.

AHR, albumin–high-density lipoprotein cholesterol ratio; AHM, albumin multiplied by high-density lipoprotein cholesterol; ALT, alanine aminotransferase; AST, aspartate aminotransferase; CHD, coronary heart disease; Cr, creatinine; GLU, glucose; HbA1c, glycosylated hemoglobin; T2DM, type 2 diabetes mellitus.

### Subgroup analysis

**Figure 5:**
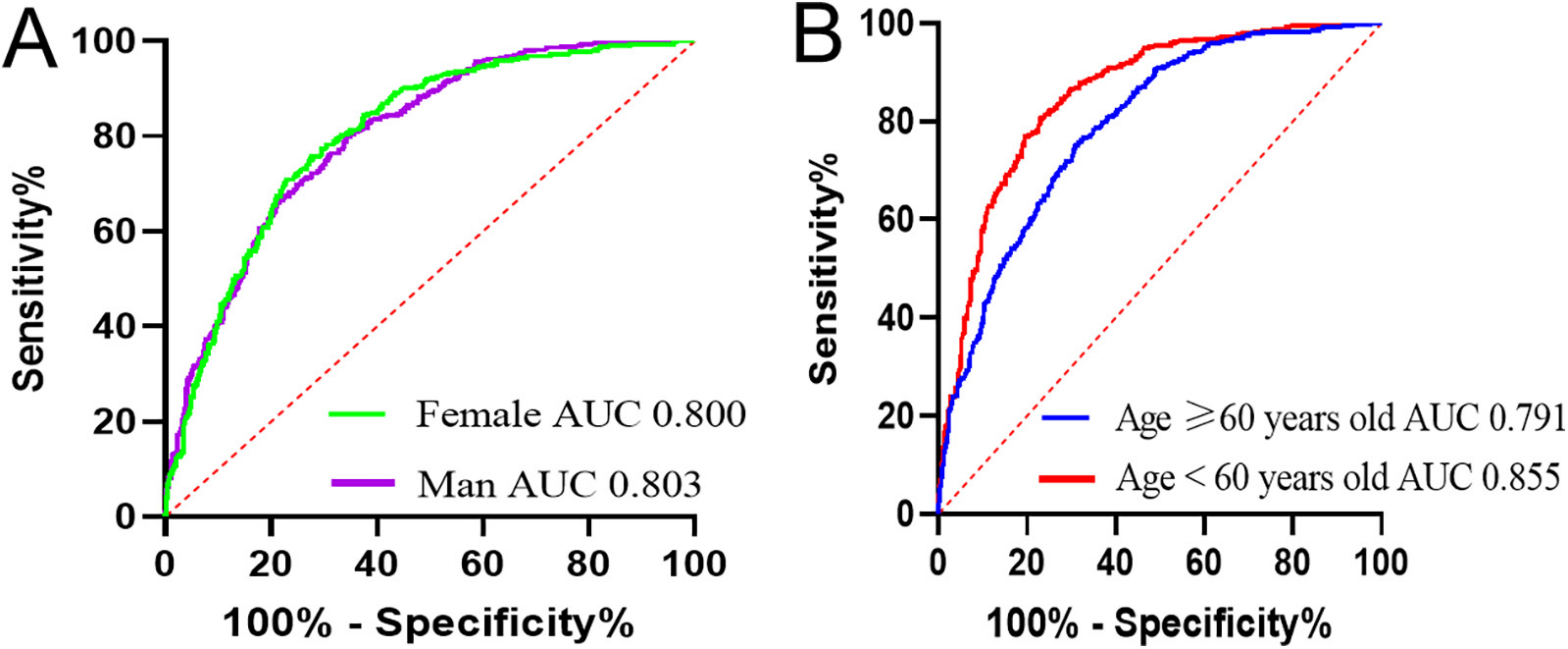
Receiver operating characteristic curves of AHM for identifying CHD in different subgroups. AHM, albumin multiplied by high-density lipoprotein cholesterol; CHD, coronary heart disease. **(A)** Based on sex. **(B)** Based on age.

The patients were stratified into four groups based on sex and age: male, female, <60 years, and ≥60 years. Univariate and multivariate analyses identified AHM as an independent risk factor for CHD across all groups (Table 6). The AUC for AHM among female patients [0.800 (95% CI: 0.775–0.825, *P* < 0.001)] closely matched that of male patients [0.803 (95% CI: 0.777–0.829, *P* < 0.001)]. In patients under 60 years, AHM exhibited stronger diagnostic performance, with an AUC of 0.855 (95% CI: 0.830–0.881, *P* < 0.001), compared with 0.791 (95% CI: 0.768–0.815, *P* < 0.001) in those aged 60 or older (Figure 5). These findings suggest that AHM may be most predictive for CHD in patients younger than 60.

**Table 6:** Univariate and multivariate logistic regression analyses in different subgroup for CHD patients.

	Univariate		Multivariate	
	OR (95% CI)	*P*	OR (95% CI)	*P*
Sex				
Male	0.913 (0.901–0.925)	<0.001	0.913 (0.890–0.936)	<0.001
Female	0.922 (0.912–0.932)	<0.001	0.904 (0.880–0.928)	<0.001
Age (year)				
<60	0.893 (0.879–0.908)	<0.001	0.896 (0.867–0.925)	<0.001
≥60	0.926 (0.918–0.935)	<0.001	0.887 (0.869–0.905)	<0.001

After adjusting for confounding factors: sex, age, hypertension, T2DM, smoking, drinkers, LDL-c, TG, TC, ALT, AST, HbA1c, GLU, Cr, UA.

ALT, alanine aminotransferase; AS, arteriosclerosis; AST, aspartate aminotransferase; Cr, creatinine; GGT, glutamyl transpeptidase; GLU, glucose; HbA1c, glycosylated hemoglobin; LDL-c, low-density lipoprotein cholesterol; ROC, receiver operating characteristic; TC, total cholesterol; TG, triglycerides; T2DM, type 2 diabetes mellitus; UA, uric acid.

## Discussion

Through the development of a multivariate logistic regression model, this study identified male gender, hypertension, T2DM, and smoking as independent risk factors for CHD. In contrast, higher levels of HDL-c, ALB, and AHM were significantly associated with a reduced risk of CHD, highlighting their protective roles. After controlling for confounding variables, multivariate logistic regression identified AHM as an independent protective factor against CHD. ROC curve analysis demonstrated that AHM achieved an AUC of 0.808 (95% CI: 0.791–0.825), significantly outperforming other indicators in diagnosing CHD. The GS score, a validated angiographic scoring system, has been widely used to quantify the severity of coronary lesions in CHD. Notably, we observed a significant negative correlation between AHM and GS, suggesting its potential role in early risk stratification for CHD patients. Subgroup analyses indicated that AHM conferred the highest predictive accuracy for CHD among individuals under 60 years, highlighting its age-specific utility.

CHD is a common CVD, and its natural history usually begins with AS, a chronic, lipid-driven inflammatory arterial disease (24). Abnormal lipid metabolism is one of the major risk factors for AS (25). Therefore, it is meaningful to evaluate serum lipid profiles in patients with CHD. Previous studies have confirmed that HDL-c is negatively correlated with CHD (26, 27). The landmark Framingham study showed that compared with high HDL-c levels, low HDL-c levels increased the risk of CHD. It also confirmed a strong negative correlation between HDL-c levels and CHD risk, with HDL-c being the strongest predictor among the evaluated lipid risk factors (28). Gu et al. (29) analyzed six large prospective studies and found that patients with CHD exhibited significantly lower HDL-c levels compared with those of participants in a case–control study. Among 267,500 Chinese participants, lower HDL-c concentrations (<50 mg/dl, 1.3 mmol/L) were significantly associated with an increased risk of stroke. Notably, each 1 mmol/L increase in HDL-c was associated with a 16% reduction in ischemic stroke risk and a 21% reduction in hemorrhagic stroke risk. Our study found that HDL-c levels were significantly lower in the CHD group compared with those in the control group, with HDL-c demonstrating a significant negative correlation with GS scores (*P* < 0.05). Univariate logistic regression analysis identified HDL-c as a protective factor against CHD. This protective effect likely stems from structural and functional impairments of HDL-c, which are induced by acute and chronic inflammation in CHD patients. Apolipoprotein A-1 is an important component of HDL-c, primarily responsible for lipid transport and metabolism (30, 31). During inflammation, serum amyloid A (SAA) levels rise and displace apoA-1 from HDL particles, impairing cholesterol efflux and promoting the entrapment of HDL in arterial walls. This process exacerbates oxidative modification of HDL particles, diminishing their anti-atherosclerotic capacity by impairing cholesterol efflux and antioxidant functions (32).

ALB is an important protein present in human plasma. The liver is the only site of ALB synthesis, and its production is regulated by factors such as nutritional status, inflammation, osmotic pressure, and hormones (12). ALB performs diverse physiological roles, including maintenance of plasma colloid osmotic pressure, transport of hydrophobic substances (e.g., bilirubin, fatty acids, and drugs), and exertion of anti-inflammatory and antioxidant activities (33, 34). Hypoalbuminemia may increase blood viscosity and compromise vascular endothelial function, thereby potentially predisposing to ischemia–reperfusion injury through impaired microvascular perfusion. These effects—combined with increased thrombotic propensity and enhanced inflammatory vulnerability—likely contribute to the pathogenesis of AS by promoting lipid deposition, vascular inflammation, and plaque instability (35). Vincent et al. (36) demonstrated that individuals with declining serum ALB concentrations—even when levels remained within the normal reference range—had a significantly elevated risk of CVD. Serum ALB levels are significantly inversely correlated with CVD incidence and progression. Clinical data show that lower ALB concentrations are associated with more severe coronary artery disease, with ALB levels decreasing progressively as disease severity increases (37). A 15-year cohort study found that hypoproteinemia was an independent predictor of cardiovascular and cerebrovascular disease (38). The results showed that serum ALB levels were significantly lower in the CHD group than those in the control group (*P* < 0.05), consistent with previous findings.

In summary, the well-documented negative association between ALB, HDL-c, and CHD reflects their roles in inflammation–nutrition balance and vascular protection. The synthesis and excretion of these proteins are intricately regulated by nutritional status (e.g., amino acid availability for ALB), inflammatory cytokines (e.g., IL-6 suppression of ALB and HDL dysfunction), and metabolic hormones (e.g., thyroid hormone modulation of HDL biogenesis). Understanding these regulatory networks may uncover novel therapeutic targets for CHD prevention. HDL-c levels are inversely correlated with conditions such as chronic renal failure, T2DM, and liver cirrhosis, reflecting dysregulated lipoprotein metabolism in these diseases (39). Malnutrition, inflammation, diabetes, liver disease, and infection can reduce albumin production, thereby leading to hypoalbuminemia. Each factor exerts distinct effects (40). Thus, it warrants clinical consideration that limitations exist in evaluating CHD risk using HDL-c or ALB alone, given their susceptibility to confounding factors such as inflammation, nutrition, and comorbidities. Therefore, we have developed a new composite indicator, the atherosclerosis heart marker (AHM), and analyzed its sensitivity and specificity for the diagnosis of CHD. This study introduces AHM as a novel composite indicator integrating serum ALB (a nutritional marker) and HDL-c (a lipid parameter), potentially providing broader predictive value than individual biomarkers. Univariate and multivariate analyses consistently identified AHM as a significant predictor of CHD risk among the examined factors. AHM was an independent risk factor for CHD. The area under the ROC curve (AUC) for AHM was significantly higher than that for HDL-c (0.808 vs. 0.777, *P* < 0.001). To assess the association between AHM and coronary artery stenosis severity, we used the GS score system to quantify lesion severity. AHM showed a negative correlation with GS scores and emerged as an independent risk factor for multivessel CHD. These findings demonstrate AHM's utility in quantifying coronary artery stenosis, as evidenced by its strong negative correlation with GS scores (Pearson *r* = −0.150, *P* < 0.001) and higher diagnostic accuracy than single markers.

## Strengths and limitations

Our study conferred several strengths. First, we for the first time elucidated the potential of AHM in identifying CHD, establishing its significant negative correlation with coronary stenosis severity. As a readily accessible biomarker from routine blood tests, AHM has the potential to enable clinicians to detect CHD at earlier stages and refine risk stratification, supported by its high sensitivity (75.7%) for preclinical atherosclerosis. Second, all patients underwent gold-standard CAG, with CHD diagnosed based on ≥50% luminal stenosis; those initially diagnosed by coronary computed tomography angiography (CCTA) were excluded to minimize diagnostic bias. However, this study also had limitations. First, the retrospective design inherently resulted in incomplete laboratory data for certain patients—particularly lipid profiles and inflammatory markers—and unmeasured confounders (e.g., family history, socioeconomic status, and lifestyle factors) were not adjusted for in the multivariate analysis, potentially introducing residual confounding bias. Second, current studies lack specific clinical reference values for AHM in assessing CHD and coronary stenosis severity. Large-sample studies are needed to establish risk assessment thresholds, thereby enabling precise risk stratification and optimized management of CHD patients. Finally, the study's single-center design and small sample size represent limitations. Future large-scale, multicenter prospective trials are needed to validate the clinical utility of this indicator and establish population-specific reference ranges for AHM.

## Conclusion

AHM was significantly linked to an elevated risk of CHD. The lower the AHM level, the greater the CHD occurrence rate. AHM is associated not only with the occurrence of CHD but also with the severity of coronary artery stenosis. The ratio serves as a practical clinical indicator, combining accessibility, cost-effectiveness, minimal invasiveness, and widespread adoption. Its clinical value lies in facilitating early CHD detection, disease severity assessment, timely intervention, and prognostic improvement, making it suitable for broader clinical implementation.

## Data Availability

The original contributions presented in the study are included in the article/Supplementary Material; further inquiries can be directed to the corresponding author.
